# Monitoring the Detailed Dynamics of Regional Thermal Environment in a Developing Urban Agglomeration

**DOI:** 10.3390/s20041197

**Published:** 2020-02-21

**Authors:** Yue Liu, Hui Li, Peng Gao, Cheng Zhong

**Affiliations:** 1 Three Gorges Research Center for Geo-hazard, Ministry of Education, China University of Geosciences, Wuhan 430074, China; yueliu@cug.edu.cn; 2 School of Earth Sciences, China University of Geosciences, Wuhan 430074, China; rslihui@cug.edu.cn; 3 Department of Geography, University of South Carolina, 709 Bull St., Columbia, SC 29208, USA; gaop@mailbox.sc.edu

**Keywords:** urban agglomeration, regional thermal environment, urban heat island, urbanization

## Abstract

Many studies have revealed the characteristics and spatial-temporal dynamics of the thermal environment in specific cities or urban agglomerations (UA), as well as the associated determining factors. However, few studies focus on the changing relationships (the difference, distance, interaction, etc.) among inner cities’ heat islands in a UA, which represent not only the detailed dynamics of regional thermal environment (RTE), but also the changing competition and cooperation among cities in a developing UA. In this study, we used Moderate Resolution Imaging Spectroradiometer (MODIS) land surface temperature (LST) products to map and analyze the detailed dynamics of the Beijing-Tianjin-Hebei (BTH) UA thermal environment. From 2001 to 2015, the mean surface urban heat island intensity (SUHII) of the BTH increased significantly, and the surface urban heat islands (SUHIs) in the southern BTH have rapidly increased, expanded and connected, eventually forming a large heat islands agglomeration. According to correlation analysis, urban sprawl probably led to the expansion and enhance of SUHIs in the south plain, while the forest has significantly alleviated urban heat island effect in northern mountains. The results expose the detailed evolution process of BTH thermal environment, and the changing relationships among the inner cities. In a developing UA, mitigation solutions (e.g., ecological corridors or controlling energy consumption) are in demand to stop the formation of a great heat region.

## 1. Introduction

The “urban agglomeration (UA)”, represents groups of cities with compact spatial organization and close economic connections. An UA includes at least one core city (the biggest and most important one) and some satellite cities, and they tend to have ordered and clear hierarchy and division of functions (industry, commercial, or entertainment) [1]. UAs are becoming the engines of global economy, as they actively engage in global competition, cooperation, and exchange of capital, information, and labor. Although the 43 largest UAs occupy very small land areas and hold less than 20 percent of global population, they will account for approximately 66 percent of global economic activities and more than 80 percent of technological innovations by 2050 [2]. Recently, China has invested greatly to develop and promote its UAs economically and politically, so that they have gradually become global economic centers [3]. The rapid development of the top UAs in China has caused serious eco-environment problems, including natural landscape loss, greenhouse gas emissions, urban heat island (UHI), air and water pollution, etc., which have attracted national and global concerns [4,5,6]. 

Higher temperatures in urban areas (namely UHI) will accelerate the accumulation of harmful gases and smoke, and greatly increase the occurrence of digestive, nervous or respiratory system diseases, such as bronchitis, emphysema, asthma, sinusitis, pharyngitis, etc. [7,8,9]. Coupled with the UHI effect, heat waves will become more frequent, stronger and longer in the climate change context, and then exacerbate serious threat to urban residents [10]. Reference [11] found inhabitants of urban areas may experience sustained thermal stress both day and night, which may result in heat-related mortality in urban regions. In a warming and urbanizing world, the growing need is not negligible to protect the urban population by increasing awareness and preparedness against thermal risk [12].

Remote sensing has been widely used for investigating urban thermal environment and the associated drivers during the urbanization process, as it can quickly and frequently monitor large area surface change with lower cost, compared to filed survey [13,14,15]. Many studies have revealed the characteristics and spatial-temporal dynamics of the thermal environment in specific (big) cities, and the related driving factors [16,17,18,19,20]. However, the characteristics and dynamics of UAs’ thermal environment remain poorly understood [21,22,23]. Many researches have proved that just taking core cities into account, while omitting other inner cities of an UA, would most likely result in underestimation of the UHI effect [24,25]. Thus, the thermal issue of an UA should be analyzed and planned from a regional perspective, not a single city view. 

Reference [25] found the UHI effect generated around Beijing and Tianjin shown complex interactions with local thermal circulations. Specifically, the UHI circulation around Beijing breezed at the southern slopes of Yan Mountain, while in the coastal area, the increased land/sea temperature gradient considerably accelerated the sea breeze along Bohai Bay. In reference [26], the influence of land cover change on the UHI dynamics in the Beijng-Tianjing-Hebei (BTH) area was investigated. Heat islands in the area were significant increased due to the transfer from natural lands (forest and cultivated lands) to inhabited lands. Reference [27] found from 1995 to 2015, the isolated urban heat islands were gradually connected and interacted with each other, forming a regional heat island in the Pearl-River-Delta (PRD) region, where built-up land increased significantly, while the ecological land was dramatically reduced. Reference [28] found that the UHI of an UA influenced range increased from 0.45 to 2.23 °C, including the interior of the UA and its surface urban heat island footprint (FP), and 23% of the influence on the RTE was caused by fringe areas. Reference [29] found that urbanization warmed the land surface regardless of urban area size in YRD area, and the UHI intensity varied markedly by cities, yet the largest did not happen in the presumed core cities. The spatial-temporal patterns depend strongly on the background climate (precipitation and air temperature), vegetation activity, surface albedo, and population density, with contrast mechanisms during the day and night. Reference [30] indicated that population density, energy consumption, average temperature and urban area had a significant positive correlation with UHI intensity; however, the average wind speed and average precipitation were significantly negatively correlated with it. Reference [31] demonstrated UHI effect within Chinese three major UAs (the BTH, YRD and PRD) was serious during 2010–2014, and the morphology of heat islands was significantly influenced by urban expansion. Urban population and electricity consumption were the socioeconomic factors that exerted the greatest influence on the size of heat islands in China’s major UAs. 

However, few studies focus on the changing relationships (the difference, distance, interaction, etc.) among inner cities’ UHIs, which reflects the detailed RTE dynamics, and probably the changing competition and cooperation among inner cities along with the UA’s development. This study aims to: (1) quantify the annual dynamics of an UA’s thermal environment and the influences of the associated determining factors; and (2) reveal the changing difference, correlation, and interaction among the inner cities’ thermal environment within the UA. Through this work, it is expected to explore the detailed evolution process and driving mechanism of the UA’s thermal environment.

## 2. Materials and Methods

### 2.1. The Study Site

**Figure 1:**
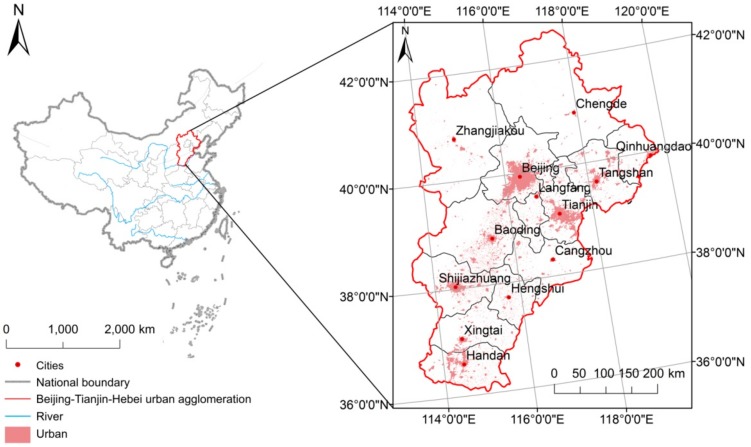
The study site.

This study was conducted in the BTH region, one of the most developed and populated UA in China [32], as shown in Figure 1. The region is mainly located in the North China Plain (115°25′E–119°19′E, 38°42′N–41°03′N), accounting for a land mass of 48,197 km^2^. The BTH area has a monsoon-influenced humid continental climate, characterized by hot, humid summers due to the East Asian monsoon, and cold, windy, dry winters influenced by the vast Siberian anticyclone. The yearly rainfall ranges between 300 mm and 800 mm, the yearly mean temperatures fluctuates from 4 °C to 17 °C. The northern part of the BTH is mountainous (near the Taihang Mountains and the Yanshan Mountains), with an altitude ranging from 200 m to 1,000 m. The southern part of the BTH area is the North China Plain close to the Bohai Sea.

In recent decades, the BTH region has experienced rapid economy development, urban expansion and population immigration, which result in serious air pollutions and urban heat islands [33,34]. Studies focusing on specific cities (Beijing, Tianjin) have been carried out for alleviating the UHI within each city [35,36]. However, investigations from a regional perspective are still necessary to better understand and reduce the regional thermal problems.

### 2.2. Data and Preprocessing

#### 2.2.1. MODIS Products

MODIS products have been successfully used to conduct thermal environment study in BTH area [31,32,33]. Hence, the monthly 1-km MODIS LST products were selected to investigate the RTE dynamics in this study. In order to save time, we downloaded the same MODIS products from the Chinese Geospatial Data Cloud (http://www.gscloud.cn/), rather than the NASA website. Images partially covering this region must be merged to cover the whole area of the BTH. Besides, the original sinusoidal projection has to be adjusted to match that of other data. Both the image merging and reprojecting were conducted in the MODIS Reprojection Tool. Then, noise caused by cloud or fog were filtered with the band math tool of ENVI 5.4. The left blank pixels were then repaired with the kriging interpolation. Finally, yearly LST images were produced by averaging the monthly LST products, and used for analyzing the yearly dynamics of the BTH thermal environment later.

MODIS composite land-cover products (MOD12Q1, MCD12Q1) were selected to delimit the urban area. They were downloaded from the official website of NASA’s Land Processes Distributed Active Archive Center (https://lpdaac.usgsgov/data_access/data_pool), as they are not available in the Geospatial Data Cloud. The products were then rescaled from 500 m to 1000 m, to match the resolution of the other data. 

The monthly MODIS NDVI products (MOD13A3) at a 1-km resolution were also downloaded from the Chinese Geospatial Data Cloud (http://www.gscloud.cn/). They were used to analyze the influence of vegetations on RTE in this study.

#### 2.2.2. Night-Time Light Data

As an important indicator of human activity, The night-time light (NTL) data has been successfully implemented in urbanization and thermal environment studies [37,38,39,40]. The Operational Linescan System (OLS) sensors of DMSP provides the longest time series NTL data, dating back to 1992 [40]. The sensors are capable of detecting weak inner-city lights even in small-scale built-up regions. In this study, the DMSP/OLS data at a 500 m resolution was used to analyze the influence of human activity on RTE. The data was downloaded from the Chinese Geographic Information Monitoring Cloud (http://www.dsac.cn/), and then rescaled to 1000 m. 

### 2.3. Methods

#### 2.3.1. The Overall Dynamics of BTH Thermal Environment

At first, the spatial-temporal dynamics of BTH thermal environment from 2001 to 2015 were mapped, in terms of yearly surface urban heat island (SUHI) distribution. Then, the general tendency was further analyzed with the Mann-Kendall (M-K) test.

SUHI represents the phenomenon that the urban surface temperature is higher than that of surrounding suburbs. As an important feature of urban thermal environment, its spatial-temporal dynamics can suggest the development process of an UA’s thermal environment [35]. Here, the SUHI intensity (SUHII) is defined as the difference between the urban surface temperature and adjacent suburban surface temperature [36]:

(1) $$SUHII_{i}=T_{i}-T_{sububs}$$

where $T_{i}$ represents the temperature of an urban pixel, and $T_{\mathrm{sububs}}$ indicates the mean temperature of suburban area. The MODIS land-cover product shows the urban region and suburbs (Figure 1). 

In the M-K test, the original hypothesis H_0_ indicates no evident trend, while the optional H_a_ suggests an evident trend (upward or downward). Below statistic is used for the series data *X*_1_, …, *X_n_* [37].

(2) $$S=\sum_{k-1}^{n-1}\sum_{j=k+1}^{n}Sgn\left(X_{j}-X_{k}\right)$$

with

(3) $$Sgn\left(X_{j}-X_{k}\right)=\left\{\begin{array}{c} \begin{array}{cc} +1 & \left(X_{j}-X_{k}\right)>0 \end{array}\\ \begin{array}{cc} 0 & \left(X_{j}-X_{k}\right)=0 \end{array}\\ \begin{array}{cc} -1 & \left(X_{j}-X_{k}\right)<0 \end{array} \end{array}\right\}$$

If S > 0, later measures is like to be greater than earlier observations; if S < 0, the overturn is right. S is calculated by:

(4) $$V_{\alpha r}(S)=n(n-1)(2n+5)/18$$

If n > 10, below statistic is used for M-K test:

(5) $$Z=\left\{\begin{array}{cc} \frac{S-1}{\sqrt{V_{ar}\left(S\right)}} & S>0\\ 0 & S=0\\ \frac{S-1}{\sqrt{V_{ar}\left(S\right)}} & S<0 \end{array}\right\}$$

Given a level of confidence alpha α, if $\left|Z\right|\geq Z_{1-\alpha /2}$, the hypothesis H_0_ is unacceptable, which means a monotonic upward or downward trend exists in the data. If Z > 0, the trend of the data is upward; if Z < 0, the trend is downward.

#### 2.3.2. The Changing Relationship among Inner UHIs

In order to discover the changing relationships (the difference, distance, interaction, etc.) among inner cities’ heat islands, a two-step process was designed in this study. At first, a landscape pattern analysis on the BTH thermal environment was conducted to find the changing aggregation and connectedness among inner SUHIs; then, the same analysis was implemented on typical cities’ UHI to find the changing differences among them. Three indices suitable to investigate the difference, aggregation and connectedness level, were involved, including the percentage of landscape (PLAND), the cohesion index (COHESION), and the proportion of like adjacencies (PLADJ).

1. PLAND
  PLAND indicates the area proportion of a specific land type [38].
  (6) $$\mathrm{PLAND}=\frac{\sum_{j=1}^{n}a_{ij}}{TA}\times 100$$
  where $a_{ij}$ indicates the area of the patch ij, i represents the land type of a patch, and *TA* represents the total land area. PLAND equals to 100 percent if a single patch covers the entire study region. It equals to 0 if a specific patch type rarely exists.
2. COHESION
  COHESION measures the level of connectedness of a specific land type [38].
  (7) $$COHESION=\left[1-\frac{\sum_{j=1}^{n}p_{ij}}{\sum_{j=1}^{n}p_{ij}\sqrt{a_{ij}*}}\cdot {\left[1-\frac{1}{\sqrt{TA*}}\right]}^{-1}\right]\times 100$$
  where $a_{ij}$ represents the area of the patch ij, $p_{ij}$ indicates the perimeter of patch *ij*, and TA indicates the number of patches.
  COHESION approaches 0 when the share of the specific type goes down, becomes less connected and further subdivided. The indicator goes up when the share of the specific type goes up, and turns more connected and aggregated.
3. PLADJ
  PLADJ measures the level of clump of the specific land type [38].
  (8) $$\mathrm{PLADJ}=\frac{\sum_{i=1}^{m}g_{ii}}{\sum_{i=1}^{m}\sum_{k=1}^{m}g_{ik}}\times 100$$
  where $g_{ii}$ indicates the number of similar joins between cells belonging to land type *i*, and $g_{ik}$ represents the number of joins between cells belonging to land type *i* and *k*. It approaches 0 if the land type is minimally clumped and no similar joins exists. PLADJ equals 100% if a single patch occupies the study area.

#### 2.3.3. Analysis on Driving Mechanism

It is commonly believed that the vegetation coverage, impervious surface, and economic development have significant influence on the UA thermal environment [39,40,41]. In order to discover the driving mechanism of the detailed dynamics of BTH thermal environment, the Spearman’s correlation coefficient was employed to investigate the influence of possible driving factors with hundreds of randomly selected samples.

1. The vegetation fractional coverage (VFC)

Vegetation, consisting of crop, grass, forest and etc., plays very important role in the ecological environment [42,43,44]. The vegetation fractional coverage (VFC), in terms of the percentage of the vertical projected area of vegetation (including leaves, stems and branches) on the ground in the total area, has been widely employed to evaluate the status and performance of eco-environment. The below formula for VFC is employed in this study [45]:

(9) $$\mathrm{VFC}=\frac{{\mathrm{NDVI}-\mathrm{NDVI}}_{\mathrm{soil}}}{{\mathrm{NDVI}}_{\mathrm{veg}}-{\mathrm{NDVI}}_{\mathrm{soil}}}$$

where NDVI is the normalized difference vegetation index; ${\mathrm{NDVI}}_{\mathrm{veg}}$ indicates the NDVI value of a pixel fully covered by vegetation; while ${\mathrm{NDVI}}_{\mathrm{soil}}$ represents the NDVI value of a pixel fully covered by bare soil. Generally speaking, ${\mathrm{NDVI}}_{\mathrm{veg}}$ and ${\mathrm{NDVI}}_{\mathrm{soil}}$ are considered as the maximum and minimum values in an NDVI image, respectively. In this study, the 5% and 95% levels in the percentile rank of all NDVI values were selected as ${\mathrm{NDVI}}_{\mathrm{soil}}$ and ${\mathrm{NDVI}}_{\mathrm{veg}}$, respectively, to avoid the influence of extreme values. 

2. The impervious surface fraction (ISF)

Impervious surfaces represent the natural or man-made impermeable ground surfaces that prevent water from penetrating into the ground, such as roofs, asphalt or concrete roads, and parking lots [46]. Based on the vegetation-impervious surface-soil (VIS) model, the ISF and the VFC vary inversely in urban area, and the formula for calculating ISF can be defined as below [47]:

(10) $$ISF=1-{\left(\mathrm{VFC}\right)}^{2}$$

Here, VFC indicates vegetation fractional coverage, and can be figured out with Equation (9).

3. The economic development indicator 

Studies have revealed that the night time light (NTL) is significantly positively correlated with human economic activities (population, GDP, electric power consumption, etc.) at regional and global scales [48,49]. Hence, NTL was used as an typical economic development indicator, to analyze the influence of human activity on RTE. 

4. Spearman’s Correlation Coefficient 

Spearman’s correlation coefficient can be used to investigate the relationship of two variables (*X* and *Y*), whose observations are paired grade data, regardless of their distribution and sample size. Sort *X* and *Y* (in ascending or descending order synchronously) to get two ranking sets *x* and *y*. The correlation coefficient between *X* and *Y* can be calculated by *x*, *y* as follows [50]:

(11) $$r_{xy}=\frac{\sum_{i=1}^{n}\left(x_{i}-\bar{x}\right)\left(y_{i}-\bar{y}\right)}{\sqrt{\sum_{i=1}^{n}{\left(x_{i}-\bar{x}\right)}^{2}}\sqrt{\sum_{i=1}^{n}{\left(y_{i}-\bar{y}\right)}^{2}}}$$

where the elements *x_i_* and *y_i_* are the rank of *X_i_* in *X* and the rank of *Y_i_* in *Y*, respectively; $\bar{x}$ and $\bar{y}$ indicate the mean value of each variable, respectively. The value range of Spearman’s *r* is [−1, +1], where 1 indicates a strongest positive correlation, −1 means a strongest negative correlation, while 0 indicates there is no correlation between *X* and *Y*.

## 3. Results

### 3.1. The Overall Dynamics of the BTH Thermal Environment

**Figure 2:**
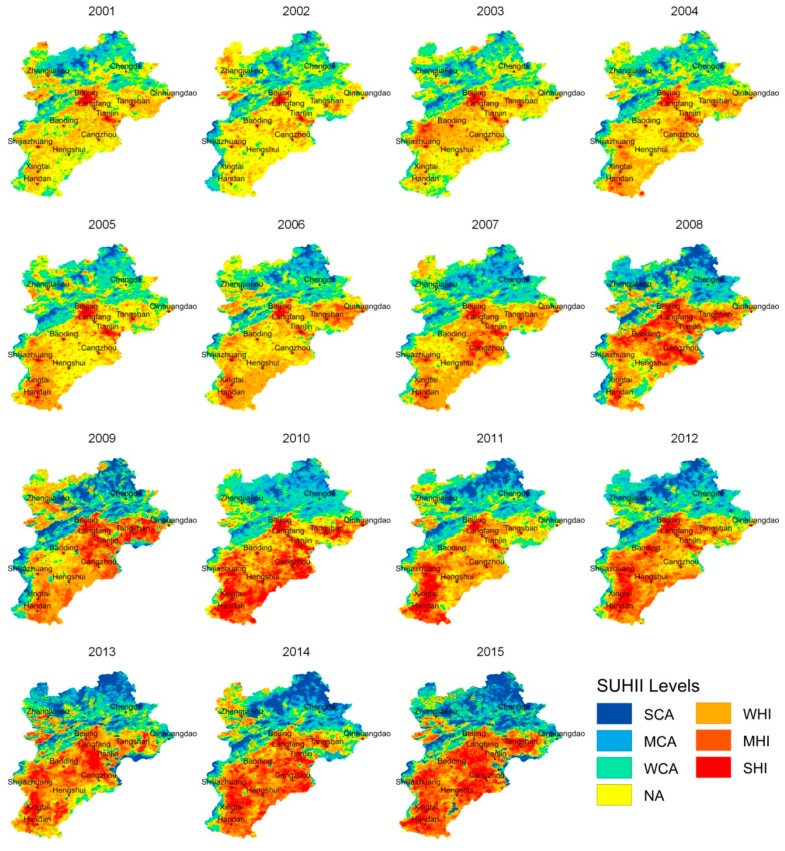
The overall dynamics of the Beijing-Tianjin-Hebei (BTH) thermal environment.

At first, the overall spatial-temporal dynamics of BTH thermal environment from 2001 to 2015 were produced and shown in Figure 2, in terms of yearly mean SUHII maps. In the figure, pixels are divided into seven categories, according to their SUHII levels: (1) strong heat island (SHI, SUHII > 5 °C); (2) moderate heat island (MHI, 3 °C > SUHII > 5 °C); (3) weak heat island (WHI, 3 °C > SUHII > 1 °C); (4) normal (NA, 1 °C > SUHII > −1 °C); (5) weak cold island (WCI, −1 °C > SUHII > −3 °C); (6) moderate cold island (MCI, −3 °C > SUHII > −5 °C); and (7) strong cold island (SCI, −5 °C > SUHII).

In 2001, large strong heat islands were apparently located in Beijing and Tianjin, the core cities of this region; while other cities were covered by small strong heat islands (points). Since then, strong heat islands of all cities expanded rapidly, along with the expansion of the cities. By 2015, strong heat islands had connected with each other, forming a great regional strong heat island, which occupied the central and southern parts of the BTH area. From 2001 to 2015, the strong heat islands grew from initially covering several isolated city centers, to eventually covering more than half of the BTH agglomeration. According to further statistics, the mean SUHII of the BTH increased significantly from 2001 to 2015, at a rate of 0.1 °C/year (representing a total increase of 3.5 °C between 2001 and 2015). 

**Figure 3:**
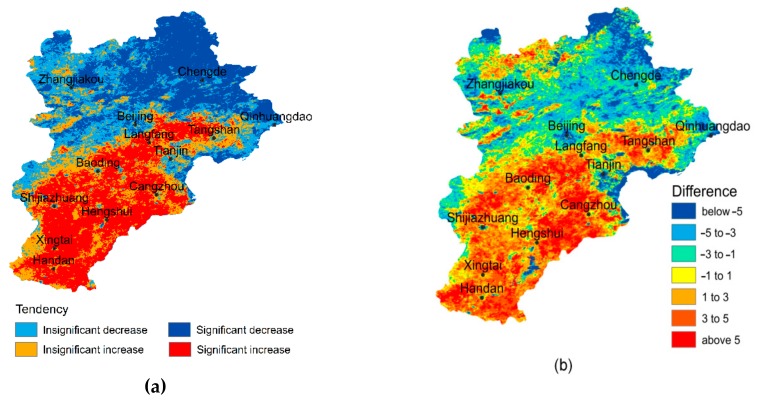
The tendency and difference of surface urban heat island intensity (SUHII) at each pixel: (**a**) The tendency of SUHII variation from 2001 to 2015, and (**b**) the difference of SUHII between 2001 and 2015. The tendency was checked with the M-K test at the 95% confidence level, and categorized into four levels, according to the Z value: 1) significant decrease (Z < −1.64); 2) insignificant decrease (−1.64 ≤ Z < 0); 3) insignificant increase (0 < Z ≤ 1.64); and 4) significant increase (Z > 1.64).

According to the M-K test results (Figure 3a), the SUHII of the southern BTH area has significantly increased, which matches the tendency of urban expansion and economic development in this region. Oppositely, the SUHII of the northern BTH area and the coast of the Bohai Sea has significantly decreased, which may be influenced by natural factors, such as forests in northern BTH, and the marine climate at the coastal plain. According to Figure 3b, the SUHII in most of southern BTH increased by more than 3 °C from 2001 to 2015, while that in most of northern BTH decreased by more than 3 °C. Both the tendency and the difference analysis reveal that the SUHII has significantly increased in the southern BTH, and evidently decreased in the northern BTH. 

### 3.2. The Changing Aggregation and Connectedness among Inner SUHIs 

Then, changing relationships between inner cities’ UHIs from 2001 to 2015were investigated with landscape metrics (e.g. PLAND, COHESION, and PLADJ).

**Figure 4:**
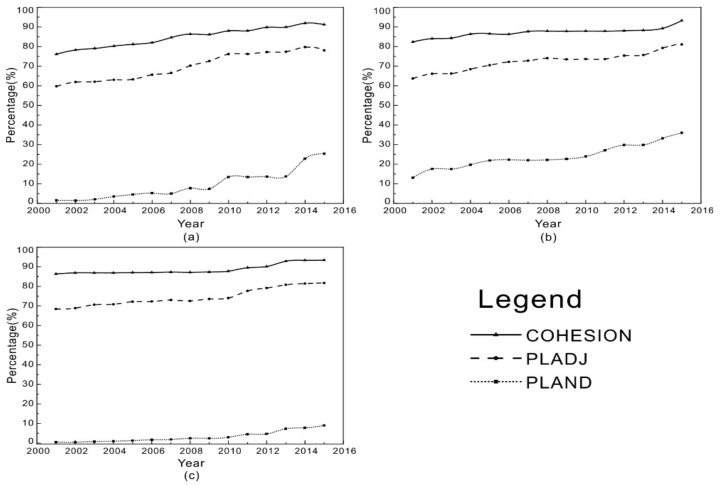
The dynamics of UHI patches: (**a**) strong heat islands; (**b**) moderate heat islands; and (**c**) weak heat islands.

According to Figure 4, the area percentages of both strong heat island category and moderate heat island category grew significantly from 2001 to 2015, at a rate of about 1.2% per year (representing an total increase of 18%), while the area percentage of the weak heat island category increased much slowly, at a rate of about 0.45% per year (representing a total increase of 6.7%). The area covered by heat islands accounted for 23.26% of BTH in 2001, then increased to 66.62% by 2015. It is worth noting that the strong and moderate heat island categories had covered 55% of BTH by 2015, suggesting a serious thermal environment threat. 

As shown in Figure 4, the PLADJ of both the strong heat island and moderate heat island categories increased rapidly from 2001 to 2015, by about 18% and 17%, respectively. The PLADJ of weak heat island category increased more slowly with several fluctuations, by about 10% over the study period. This indicates the degree of aggregation of the strong and moderate heat island category increased a lot, while that of the weak heat island category did not increase much. The COHESION of all heat island categories increased from 70% to about 90%, suggesting that the level of connectedness became very high by 2015. 

### 3.3. The Changing Differences among Inner Cities

**Figure 5:**
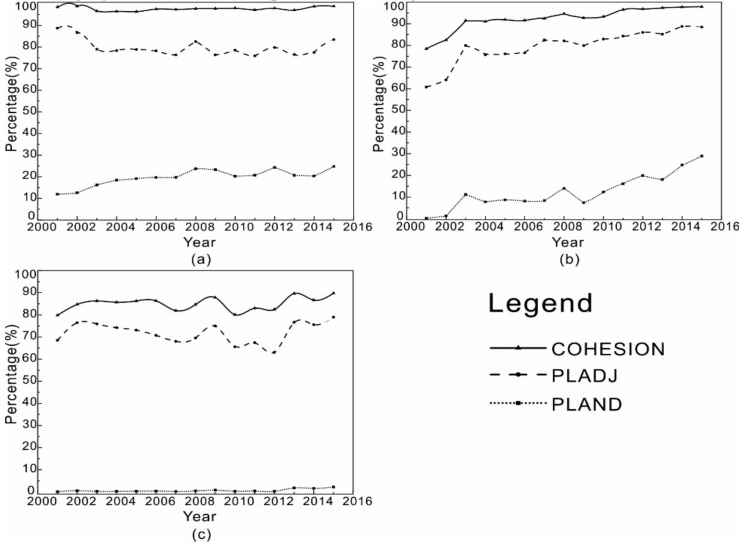
The dynamics of strong heat islands’ landscape in typical cities: (**a**) Beijing; (**b**) Shijiazhuang; and (**c**) Zhangjiakou.

Landscape analyses were then conducted on SUHIs of typical cities (e.g., Beijing, Shijiazhuang, and Zhangjiakou) to discover the difference among BTH inner cities, and shown in Figure 5. Beijing is the capital and a most developed city of China, Shijiazhuang is the capital of Hebei province and a moderately developed city, and Zhangjiakou is a developing city located in northwestern mountains. As the most serious environmental threat among all types of heat islands, the strong heat island category was selected to represent inner city’s thermal environment. 

According to Figure 5, the area percentages of strong heat island category in both Beijing and Shijiazhuang grew remarkably from 2001 to 2015, by about 12% and 20% respectively; while in Zhangjiakou it increased much slowly, by about 2% over the study period. It is worth noting that the strong heat island category occupied about 25% of Beijing’s area and 29% of Shijiazhuang’s, but only 3% of Shijiazhuang’s, as of 2015. Located in the East China Plain, Beijing and Shijiazhuang’s are well developed and their built-up areas have dramatically expanded; while the built-up area of Zhangjiakou has not expanded much, as it is located in the northwestern mountains. Besides, the UHI effect in Zhangjiakou may be significantly weakened by surrounding forests.

The PLADJ and COHESION of Shijiazhuang increased remarkably from 2001 to 2015, by about 28% and 20%, respectively, suggesting that the level of connectedness and degree of aggregation of the strong heat islands had went up apparently. The PLADJ and COHESION of Zhangjiakou increased much slowly with several fluctuations, by about 10% over the study period, indicating the level of connectedness and degree of aggregation of the strong heat islands did not rise by much. With regard to Beijing, the PLADJ and COHESION fluctuated gently during the study period, and did not change much eventually. It can be observed that the differences of all indicators between Beijing and Shijiazhuang, which were great in 2001, had almost disappeared by 2015. 

### 3.4. The Possible Driving Factors

**Figure 6:**
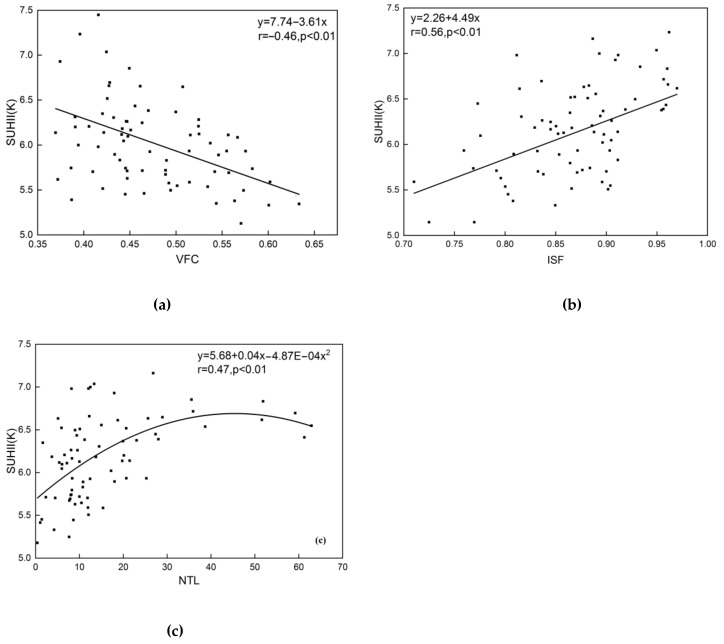
The relationships between SUHII and possible driving factors: (**a**) SUHII and vegetation fractional coverage (VFC); (**b**) SUHII and impervious surface fraction (ISF); and (**c**) SUHII and night-time light (NTL).

In order to investigate the possible driving mechanism, the Spearman’s correlation coefficients between SUHII and influence factors (e.g., VFC, ISF and NTL) were calculated and shown in Figure 6. In the test, 350 samples were stratified randomly selected in the region (50 samples for each SUHI category shown in Figure 2).

Results reveal that SUHII is significantly negatively correlated with vegetation coverage, significantly positively correlated with impervious surfaces, and less significantly positively correlated with NTL. This means vegetation could reduce the SUHII to some extent, while human activities (urban expansion and heating) will increase the SUHII. For instance, the area of UHIs in mountainous cities (e.g., Chengde and Zhangjiakou) is always small, as their economic development and urban expansion are seriously limited by steep terrain and inconvenient transports. Besides, surrounding forests can affect the transmission of solar radiation by absorbing and transferring it into biological energy, thus reducing the local SUHII [51]. Oppositely, cities in the south plain have witnessed rapid urban expansion and industrialization, benefiting from national economic policies and convenient transport systems [25,26]. Hence, their SUHIs have dramatically expanded and connected, forming a great heat region, along with the increase of impervious surfaces [52,53]. What’s worse, local SUHII cannot be effectively alleviated, as low VFC land (built-up and agriculture land) have occupied most part of the plain. It is noted that the impervious surfaces and NTL have similar influence on SUHII, as both are seriously affected by human activities. The result demonstrates that both natural (vegetation coverage) and anthropogenic factors (impervious surfaces and NTL) have a strong influence on the distribution and dynamic of regional thermal environment. 

## 4. Discussions

In recent decades, UA has become the dominant trend of global urbanization [1,2,3]. Because of rapid urbanization, UHI effects in Chinese UAs have been serious and receiving increasing attention from urban planners, researchers, and city managers. In high temperatures urban areas, smog and harmful gases will increase and accumulate, resulting in serious air pollution over the city, which will cause digestive system or nervous system diseases [7,8,9]. Studies on UA thermal environment dynamics and associated driving factors (e.g., land cover and socioeconomic factors) have been reported [25,26,27,28,29,30,31]. This study focuses on investigating the changing relationships (difference, distance, interaction, etc.) among inner cities’ UHIs in a developing UA, which could deepen our understanding of detailed RTE dynamics and the changing competition and cooperation among cities.

As municipalities directly under the Chinese Government, Beijing and Tianjin have experienced rapid economic development and urban sprawl in recent years [25,26]. Figure 2 reveals strong heat islands mainly occurred in the two cities at the beginning of this century. Figure 5 demonstrates that the differences of the area percentages, the level of connectedness and degree of aggregation of the strong heat islands between core cites (e.g., Beijing or Tianjing) and other cities were very great 2001. Since then, the Chinese authority has primarily promoted the integration and economic development of BTH, by establishing better communication networks, modern highways, and investing more educational and natural resources [54]. Promoted by economic policies, the economic of other cities in the north China plain, such as Langfang, Baoding, Handan, Xingtai, and Shijiazhuang, have also shown accelerated development during the study year [26]. The built-up regions of these cities have expanded and increased rapidly, as well as the area percentage, level of connectedness and degree of aggregation of their strong heat islands. At 2015, all the plain cities’ UHIs were connected, forming a great UHI region covering the southern BTH, and the difference among them had almost disappeared. For instance, the area percentage, level of connectedness and degree of aggregation of the strong heat island category in Beijing and Shijiazhuang became similar. Oppositely, the UHIs of mountainous cities (e.g., Zhangjiakou and Chengde) did not increase much, as their urban expansion was seriously limited by surrounding terrain and urban temperature had been significantly weakened by forests. 

It is worth noting that the regional mean SUHI had a sharp increase at 2008, which was probably caused by the preparations for the Beijing Olympic Games [55,56]. A large amount of infrastructures (i.e. stadiums, roads, hotels, etc.) were built, and millions of workers were hired for those constructions before the Games. The former led to rapid impervious surface expansion, and the latter resulted in a sharp increase in domestic heat consumption. Both factors exacerbated the UHI effect. 

Monitoring the changing relationships (e.g., difference, correlation, and interaction, etc.) among inner cities’ UHIs could investigate the complex and variable competition and cooperation among cities. This study can help understand the development history of BTH and related environmental effects, especially in thermal pollution. The serious UHI not only affects people’s life and work, but also restricts the further improvement of a city or an UA. Hence, it is very important to reduce the UHI intensity and alleviate the impact of the urban high temperature, by carefully investigating the dynamics, driving mechanism and environment influence of UHI. In this study, we also assessed the relationship between the dynamics and land use changes, socio-economic development in the BTH. The results indicate that land use changes have significant influence on UHI dynamics. Hence, in the future for urban planning and design, designers should strengthen the construction of ecological corridors to facilitate mass and energy exchange between urban areas and their surroundings. Through scientific urban layout, UHI mitigation could be achieved. Our study also reveals that UHI intensity significantly positively correlates with economic activities. To mitigate the UHI effect in the BTH, it is necessary to control the increase in the amount of energy consumption by developing new towns to disperse urban overspill, improving the combustion efficiency of fuel and advocating for public transportation. 

## 5. Conclusions

In this research, the detailed development process of BTH thermal environment was mapped and analyzed using the MODIS LST product. Overall, the mean SUHII of BTH increased gradually from 2001 to 2015, with a dramatic jump in 2008, which was probably caused by the preparations for the Olympic Games. According to detailed maps and landscape analyses, the SUHI effect in the southern BTH area has significantly increased, expanded, and connected, eventually forming a great agglomeration. Benefiting from the national policies for regional economic development, the differences among the inner cities have dramatically decreased. According to the Spearman’s correlation coefficient, urban sprawl led to the rapid expansion of the SUHI effect in the south plain, while vegetation played an important role in alleviating high temperatures in the north mountain area. The results reveal the detailed dynamics of BTH thermal environment and the changing relationships among inner cities’ heat islands, as well as possible driving mechanisms. All results may server as a scientific reference for developing better strategies for BTH sustainable development and environmental protection. In future study, more indicators or methods will be necessary to completely discover the complex relationships among inner cities’ heat islands.
